# Religious Officials' knowledge, attitude, and behavior towards smoking and the new tobacco law in Kahramanmaras, Turkey

**DOI:** 10.1186/1471-2458-11-602

**Published:** 2011-07-28

**Authors:** Mustafa Haki Sucakli, Ali Ozer, Mustafa Celik, Hasan Kahraman, Hasan Cetin Ekerbicer

**Affiliations:** 1 Department of Family Medicine, Medical Faculty, Kahramanmaras Sutcuimam University, Kahramanmaras, Turkey; 2 Department of Public Health, Medical Faculty, Inonu University, Malatya, Turkey; 3 Department of Chest Dieases, Medical Faculty, Kahramanmaras Sutcuimam University, Kahramanmaras, Turkey; 4 Department of Public Health, Medical Faculty, Kahramanmaras Sutcuimam University, Kahramanmaras, Turkey

**Keywords:** Smoking, knowledge, attitude, behavior, Imam, religious official, Turkey

## Abstract

**Background:**

Tobacco control effort should be first started in people that are looked upon as role models for the general population. We aimed to determine the knowledge, attitude, and behavior of religious officials towards smoking and the new tobacco law.

**Method:**

The study group was comprised of 492 Imams and 149 Quran course instructors working in Kahramanmaras city of Turkey, 641 religious officials in total, and our survey form was applied on 406 (63.3%) of those religious officials who agreed to participate in the study.

**Results:**

Twenty-eight (6.9%) participants were current smokers and 35 (8.6%) were ex-smokers. 99.8% of the religious officials believed that smoking was harmful and/or prohibited in terms of religion. While 43.6% respondents thought smoking was "*haram*" (forbidden by Islam), 56.2% believed it was "*makruh*" (something regarded as reprehensible, though not forbidden by God according to Islam). 85.2% of the participants were aware of the recent tobacco law. 55.5% of the respondents, who were aware of the recent tobacco law, evaluated their knowledge level on the law as adequate, whereas 44.5% evaluated it as inadequate 92.4% of the participants noted that religious officials should play active roles in tobacco control effort.

**Conclusion:**

Smoking rate among religious officials is much lower than that of general public. In order to help religious officials to take a more active role on this issue, they should be trained on the subject and appropriate platforms should be established.

## Background

Smoking is the most important preventable cause of death across the world [1]. Each year, 4 million (8.8% of the entire mortality per year) people lose their lives due to smoking-related diseases [2]. Currently, cigarette smoking is a worldwide health issue, however, while it shows a decrease in developed countries, there is an increase in cigarette smoking rates in developing countries such as ours. The elevated prevalence is observed particularly among the young people [3]. Turkey stands as the second country after Greece in terms of per capita cigarette consumption [4] and appears to be the fifth country with regard to world tobacco production [5].

As countries implement various precautions for tobacco control, international regulations on the subject are taking place, as well. Among those regulations, *Framework Convention on Tobacco Control *(FCTC) has a particular importance. This convention which was published by the *World Health Organization *(WHO) in 2003, has been designed as a treaty that compels ratifying countries to take specific legislative tobacco control actions [6]. In our country, this convention has been approved and enacted by Türkiye Büyük Millet Meclisi (TBMM) (Grand National Assembly of Turkey). In addition, Turkey has implemented the required regulations by issuing Law No. 4207 on "*Prevention of harm induced by tobacco products*" and Law No. 5727 on "*Amendments on the law for prevention of harm induced by tobacco products*" [7]. By this law, indoor smoking at public places has been prohibited completely since July 19, 2009.

The leadership of social role models (eg. healthcare workers, teachers, religious officials etc.) is an important part of a comprehensive national tobacco control effort. After succeeding in those people, they should be convinced to take active role in campaigns for smoking cessation. When we review the countries that succeeded against smoking, physicians and other workers are observed to have active roles [8,9]. If role models of the society smoke, it affects the tobacco control effort negatively. Therefore, it is imperative that first those role models should be convinced to quit smoking.

The aim of this study is to underscore the necessity of active participation of religious officials in the tobacco control effort by determining knowledge, attitude and behavior of religious officials towards smoking and the new tobacco law (NTL) in the central district of Kahramanmaras.

## Methods

Religious services in Turkey are conducted by the Directorate of Religious Affairs, and performed by mufti (head-officer of religion). In the provinces, there are mosques for prayers, with services conducted by imams, and courses on the Quran conducted by educators. The present study was performed in Kahramanmaras Province, and included officials of religion. Kahramanmaras province, located in the eastern Mediterranean region, has a population of 1 million persons, with approximately 520,000 living in the city center. In total, 641 religious officials, 492 Imams and 149 Quran course instructors, work in the central district of Kahramanmaras. Each month, the Kahramanmaras Province Religious Affairs Head-officer (mufti) holds a meeting with all of the imams in charge in the city center, and also with the Quran course instructors. Permission was obtained from the Kahramanmaras Religious Affairs Center for the present study. A 32-item questionnaire was prepared and administered to Imams and Quran course instructors during these meetings. Participants were informed about the study before the meetings on October 16 and 17, 2010. Participation was voluntary and included 406 (all attendee of the meeting) of 641 officials of religion (63.3%).

The survey aiming to assess knowledge, attitude, and behavior towards the NTL, were prepared. The questions evaluated the following characteristics and perceptions of the participants: sociodemographic status, smoking status, degree of addiction to smoking, smoking status at home, knowledge and opinion about the NTL, ruling of Islam on smoking, and thoughts about taking active part in tobacco control effort.

Standard survey forms were filled out by the participants under the supervision of the investigator. Our study was approved by the local ethics committee of our faculty.

Data were evaluated by computer with SPSS 15.0 package program for statistics. Chi-square test was used for statistical analysis. p < 0.05 was recognized as statistically significant for all values.

## Results

Mean age for 406 people who participated in the study was 37.94 ± 7.09 years (range: 21-63). Three hundred-twenty (78.8%) of the participants were male, 86 (21.2%) were female. Socidemographic characteristics of the participants are shown in Table 1.

**Table 1:** Sociodemographic characteristics of the participants (n = 406)

Sociodemographic characteristics	n	%
**Gender**		

Male	320	78.8

Female	86	21.2

**Occupation**		

Imam	312	76.8

Quran course instructor	94	23.2

**Education level**		

Primary school	3	0.7

High school	96	23.6

University	280	69.0

Master	27	6.7

**Age (year)**		

20-30	66	16.3

31-40	230	56.6

≥ 41	110	27.1

**Marital status**		

Single	29	7.1

Married	375	92.4

Widow/widower	2	0.5

Twenty-eight (6.9%) of the participants were current smokers, 35 (8.6%) were ex-smokers and 343 (84.5%) were non-smokers. In terms of gender distribution, all the current smokers were male thus smoking rate for male participants was 8.75%.

Eight point one percent of the participants had at least one smoker in their households. Most of the houses (84.8%) with smokers were houses of the non-smoker participants. Twenty-three (82.2%) of 28 smoking participants noted that they did not smoke in their houses.

About the ruling of Islam on smoking, 177 (43.6%) of the religious officials noted that it was prohibited (haram), whereas 228 (56.2%) said that it was unfavorable but not prohibited (makruh), and only one participant noted that smoking was permissible (helal) in Islam (Table 2).

**Table 2:** Knowledge, attitude, and behavior of the participants about smoking and the new tobacco law (NTL)

	Total	
Knowledge, attitude, and behavior*		
	
	**n**	**%**

**What is your smoking status? (n = 406)**		

Smoker	28	6.9

Ex-smoker	35	8.6

Non-smoker	343	84.5

**What is the ruling of Islam on smoking (n = 406)**		

Helal	1	0.2

Haram	177	43.6

Makruh	228	56.2

**Religious officials should not smoke among public (n = 406)**		

Agree	390	96.1

Disagree	16	3.9

**Religious officials should be exemplary models by not smoking cigarette (n = 406)**		

Agree	397	97.8

Disagree	9	2.2

**Smoking is an important health issue of Turkey (n = 406)**		

Agree	394	97.0

Disagree	12	3.0

**Religious officials should participate in the campaigns against smoking (n = 406)**		

Agree	375	92.4

Disagree	31	7.6

**Have you heard of the new tobacco law? (n = 406)**		

Yes	346	85.2

No	60	14.8

**Do you have adequate knowledge on the new tobacco law? (n = 406)**		

Adequate	192	55.5

Inadequate	154	44.5

Do you support the new tobacco law? (n = 346**)		

Yes, I support	326	93.4

No, I do not support	20	6.6

Where did you gain knowledge about the new tobacco law? (n = 346**)		

Television	326	94.2

Newspaper	150	43.4

Internet	61	17.6

Friend	43	12.4

Radio	25	7.2

Healthcare workers	21	6.1

In-service training	18	5.2

School	3	0.9

* There was no statistically significant difference between the Imams and Quran course instructors (p > 0.05).

**60 people who were not aware of the new tobacco law were not included.

Two hundred eighty-five (70.2%) of the participants responded that people in their parishes saw them as a role model and asked them about the ruling of Islam on smoking. Regarding the attitudes of religious officials towards their parishioners demanding help about smoking-related problems, 4.2% (n = 17) noted that they did nothing, whereas 87.4% (n = 355) told us that they informed them, 4.4% (n = 18) told that they referred them to authorized centers for further information, and 3.9% noted that they had never faced such a demand.

According to the Fagerström nicotine addiction test, 22 (78.6%) individuals were low level and 6 (21.4%) were moderate level addicts. 27 (96.4%) of those people noted that they considered to stop smoking, whereas 20 (71.4%) stated that they would like to have assistance for smoking cessation.

In terms of knowledge level of the participants about the NTL enacted in 2009, 346 (85.2%) participants were aware of it. 154 (44.5%) of those 346 people noted that they had inadequate information about the law, whereas 326 (94.2%) people said that they supported the law (Table 2).

Among the participants who were aware of the NTL, 18 had learnt it from the in-office training, whereas 326 from television, 43 from a friend, 25 from radio, 3 from a teacher, 21 from a healthcare worker, 150 from newspaper, and 61 from the internet (Table 2).

Three hundred ninety (96.1%) of the religious officials stated that they should not smoke in presence of other people and 397 (97.8%) of them said that they should constitute an exemplary model by not smoking cigarette. Moreover, 394 (97.0%) of the participants recognized smoking as an important health issue of Turkey and 375 (92.4%) of the participants said that they should take active roles in the tobacco control effort (Table 2).

Three hundred (73.9%) of the religious officials noted that they could be helpful in programs promoting smoking cessation. However, 389 (95.8%) of them stated that they had no adequate knowledge on the subject and 271 (66.7%) expressed a wish to receive training on assistance for smoking cessation.

## Dıscussıon

The influence of religion over health is taught in medical, nursing, public health, and theology schools [10]. The studies of the Eastern Asia Office of World Health Organization indicate that when used in combination with other activities, support of religious officials may benefit in prevention of some diseases and conditions [11,12]. In a study conducted by Yong et al. on smokers among Muslims in Malaysia and Buddhists in Taiwan, 61% of Muslim and 58% of Buddhist participants were found to believe that religious officials could be of assistance in smoking cessation [13]. The studies of Saeed et al. and Swaddiwudhipong et al. showed that religious officials could increase the motivation of smokers who try to quit smoking [14,15]. Another study conducted by Buddhist monks yielded similar results, as well [16]. In our study, 73.9% of the religious officials noted that they could contribute to the campaigns on smoking cessation. After establishing the appropriate platforms, religious officials can be helpful in the tobacco control effort.

Currently, nearly one third (31.2% - around 16 million) of adults older than 15 years of age smoke tobacco in Turkey [17]. In a study conducted by *Family Research Institute *and *Turkish Statistical Institute *(TUIK) in 2006, the daily smoking rate among people ≥ 18 years of age, was found to be 33.4% [18]. In terms of occupational groups, smoking rate was determined to be 41.9% for the police officers [19] and 45.8% for the physicians [20]. There are various studies focusing on smoking rates in various professions. In the study of Sezer et al., smoking rate among physicians and dentists in Elazig province was 54.9% for men and 39.5% for women. Ozturk et al. conducted a similar study on government officials in Kayseri province and found the smoking rate as 59.1%. Fidan et al. found the smoking rate among teachers in Kahramanmaras province as 32.5% [21-23]. In our study, smoking rate among religious officials in the central district of Kahramanmaras province was 6.9%. This is a significantly lower rate compared with those of the general population and other occupations. Such a low smoking rate among religious officials suggests that religion and religious officials should be utilized for maintenance of public health in terms of preventive medicine.

In the present study, 27 of 28 smoking religious officials had a wish to quit smoking, whereas 20 were observed to be ready for outside help on this subject. Therefore, authorized people tasked with assisting people in smoking cessation should be informed and those officials should be encouraged in their effort on tobacco control. Tobacco smoking is an important public health issue. In many occupations, cigarette smoking is widespread which presents negative role models for the general population [24-27]. Some of those occupational groups are more important than others in terms of smoking. Those groups which are recognized as role models due to their status (eg. physicians, teachers, artists and sportsmen) should be aware of their responsibility towards the public. In a study performed in Ankara in 1995 on occupational groups looked upon as role models, smoking rate was found to be 50.8% in teachers, 43.9% in physicians, and 34.9% in sportsmen [28]. After this date, studies including 12.500 people from various occupational groups such as drivers, artists, police officers, physicians, media workers, deputies, Imams, and muezzins, were performed in 1998 and 1999, both of which revealed a varying smoking rate between 24.8% and 74.3%. As lowest smoking rates were observed among the religious officials (24.8% in 279 male religious officials in 1999), highest rates were determined to be among drivers [29]. In another study, more than 90.0% of the participants defined religious officials as role models in terms of smoking and smoking cessation [13]. In the current study, 70.2% of the participants stated that their parishioners followed them as role models and asked questions about smoking.

In our study, among religious officials facing smoking people or individuals who require help for smoking cessation, 4.2% did nothing, whereas 87.4% informed those people about smoking, 4.4% referred them to authorized centers, and 3.9% noted having no such experience. In order to be able to assist and encourage their mosque community, religious officials themselves should have knowledge about smoking. Moreover, small number of religious officials who referred people to the authorized centers, indicates an unawareness among the religious officials that should be addressed.

In the study of Yong et al. on smokers, 79% of Muslims and 88% of Buddhists were found to believe that smoking cigarette is prohibited by their religion [13]. In the current study, 99.8% of the religious officials stated that smoking was either "haram" (prohibited by religion) or "makruh" (not forbidden, but reprehensible). Moreover, 35 (8.6%) of the participants noted that they had quitted smoking. 80% of those 35 individuals had quitted smoking within the recent decade which may be explained by the recent awareness campaigns, NTL, and recognition of smoking as "haram" or "makruh" by majority of the religious officials. As we consider the relatively higher smoking rates among healthcare workers who should be the leading role models and activists against smoking [30], participation of religious officials, among whom smoking rate is very low and smoking is believed to be prohibited by religion, in the tobacco control effort will be an important asset.

Turkey has ratified the FCTC and is taking steps to implement effective laws to control tobacco at the national level. In order to successfully execute and support this law, the government should follow a multidisciplinary and multisectoral approach by working in coordination with all the social institutions and components including non-governmental organizations, universities, mass media, and international organizations [31]. Since 94.2% of the participants in our study expressed their support towards the NTL and noted that they would readily support smoking cessation, religious officials may be counted as one of those social components, as well. Moreover, in a study conducted on physicians, their knowledge on therapies concerning tobacco addiction and the NTL, was found to be inadequate [32]. In our study, although majority of the participants (85.2%) were aware of the NTL, 44.5% of them admitted having inadequate knowledge on the law. Those results suggest that even groups that should take the leading roles in the tobacco control effort do not have adequate knowledge about the NTL.

The majority of the participants who were aware of the NTL, had heard it from the mass media (Table 2). Since the reliability of information gained from the mass media is subject to discussion, it appears that the institutions that are responsible of informing the public about the new laws should work more efficiently.

Almost all of the religious officials in our study told us that they should constitute an exemplary model by not smoking among public. Additionally, nearly all the respondents recognized smoking as an important health issue for Turkey and expressed their conviction that they should play more active roles in the tobacco control effort (Table 2). Those results indicate the responsibility felt by religious officials in the effort to control tobacco.

Twenty-two of the 28 smoking religious officials were not smoking at home although it was not prohibited. This is an indication of the fact that smoking religious officials respect the health of family members. In the current study, 95.8% of the participants stated that their knowledge was not adequate on smoking and smoking cessation, whereas 66.7% expressed a desire to be trained on smoking and assistance for smoking cessation. Therefore, religious officials should receive training in tobacco control and on the contents of the NTL so that they may assist in educating the public about tobacco control regulations and the benefits of smoking cessation. Also they should take active roles along with the healthcare workers and other supporters in the tobacco control interventions. More importantly, they should be encouraged to contribute smoking prevention programs.

### Limitations and strength of the study

All religious officials in our country are state employees and most of them are men. All religious officials attending to the monthly meeting accepted to fill out the questionnaire and attended to our study who constituted 63% of all religious officials in the city. The reason for low participation rate is the unattendance of some officials to the monthly meeting due to various excuses such as off day, authorized permission for health or being on duty. We believe that unattending officials did not affect our results, since they were not informed prior to the meeting. Thus the population in the meeting can represent all the religious officials in our city. On the other hand, these results give us information on the knowledge, attitudes and behaviors of religious officials in only one city on the NTL and this study does not represent whole Turkey. The strength of the study is the really low rate of smoking rate among religious officials, their willingness to be trained on the NTL and their capacity to access so many people.

## Conclusıon

In conclusion, due to low smoking rates and their esteemed status among public, religious officials should take an active part in smoking prevention, delivering smoking cessation counseling and smoking cessation campaigns in the leadership of healthcare professionals. Required legal framework should be built and appropriate platforms such as conducting training courses for health professionals and religious officials together could be an appropriate way of solution giving the leading role to health professionals in tobacco control.

### Ethics approval

This study was approved by the local ethics committee of our faculty. Moreover, required permissions were obtained from the Muftiat of Kahramanmaras (*council of muftis *- highest religious local authority).

## Competing interests

The authors declare that they have no competing interests.

## Authors' contributions

MHS carried out the literature review and participated in the development of the study design, data management, data analysis, interpretation of the data, writing the article and revising article drafts for publication. AO participated in the development of the study design, interpretation of the data, and revising article drafts for publication. MC had the initial idea for the study and its design and participated in the development of the study design, interpretation of the data, and revising article drafts for publication. HK participated in the development of the study design, interpretation of the data. HCE participated in interpretation of the data, and revising article drafts for publication. All authors read and approved the final manuscript.

## Funding

No funding.

## Pre-publication history

The pre-publication history for this paper can be accessed here:

http://www.biomedcentral.com/1471-2458/11/602/prepub
